# Efficacy of Ketamine with and without Lamotrigine in Treatment-Resistant Depression: A Preliminary Report

**DOI:** 10.3390/ph16081164

**Published:** 2023-08-16

**Authors:** Boney Joseph, Nicolas A. Nunez, Simon Kung, Jennifer L. Vande Voort, Vanessa K. Pazdernik, Kathryn M. Schak, Stacey M. Boehm, Brooke Carpenter, Emily K. Johnson, Grigoriy Malyshev, Nathan Smits, Daniel O. Adewunmi, Sarah K. Brown, Balwinder Singh

**Affiliations:** 1 Department of Psychiatry & Psychology, Mayo Clinic, Rochester, MN 55905, USA; 2 Department of Neurology, Mayo Clinic, Rochester, MN 55905, USA; 3 Department of Quantitative Health Sciences, Mayo Clinic, Rochester, MN 55905, USA

**Keywords:** lamotrigine, ketamine, esketamine, treatment-resistant depression

## Abstract

Intravenous (IV) ketamine and FDA-approved intranasal (IN) esketamine are increasingly used for treatment-resistant depression (TRD). Preliminary studies have suggested a synergistic effect of ketamine and lamotrigine, although the data are inconclusive. Herein, we report the response to serial ketamine/esketamine treatment among patients with TRD with or without lamotrigine therapy. In this historical cohort study, we included adult patients with TRD who received serial IV racemic ketamine (0.5 mg/kg over 40−100 min) or IN esketamine (56/84 mg) treatments. A change in depressive symptoms was assessed using the 16-item Quick Inventory of Depressive Symptomatology self-report (QIDS-SR) scale. There were no significant differences in response or remission rates among the patients on or not on lamotrigine during the ketamine/esketamine treatments. For a percent change in the QIDS-SR from baseline, no interaction was found between the lamotrigine groups and treatment number (*p* = 0.70), nor the overall effect of the group (*p* = 0.38). There was a trend towards lower dissociation (based on the CADSS score) among current lamotrigine users, especially in patients who received IV ketamine. A major limitation is the limited number of patients taking lamotrigine (n = 13). This preliminary study provides insufficient evidence that continuing lamotrigine therapy attenuates the antidepressant effect of repeated ketamine/esketamine; however, there seems to be a signal toward attenuating dissociation with lamotrigine in patients receiving serial ketamine treatments. Further observational studies or randomized controlled trials are needed to replicate these findings.

## 1. Introduction

Ketamine, a novel rapid-acting antidepressant, is an N-methyl-D-aspartate (NMDA) receptor antagonist thought to increase the release of synaptic glutamate [1,2,3]. Ketamine has a short half-life with a brief duration of antidepressive effects lasting for a few days [4]. Often, patients with treatment-resistant depression (TRD) who respond to ketamine require serial infusions and maintenance treatment to sustain the antidepressant response [5]. So far, there are limited data regarding therapeutic interventions that can prolong or enhance ketamine’s short-acting antidepressant effects.

Lamotrigine is a voltage-sensitive sodium channel inhibitor, a glutamate modulator, and a potential glutamate release inhibitor [6]. It is also a mood-stabilizing agent with antidepressant properties [7]. Recent pre-clinical animal studies have suggested synergistic effects of lamotrigine and ketamine, although, the data are equivocal [8]. In a small randomized controlled trial (n = 16), Anand et al. showed that pretreatment with 300 mg of lamotrigine significantly decreased ketamine-induced perceptual abnormalities and enhanced the rapid mood-elevating effects of ketamine in healthy adults [9]. In another study investigating the effect of intravenous (IV) ketamine on regional blood oxygenation level-dependent (BOLD) signals in healthy men, the authors reported attenuation of most BOLD signals and lower dissociation with 300 mg of lamotrigine pretreatment [10]. However, a subsequent study conducted in patients with TRD failed to replicate these results [11]. Pretreatment with lamotrigine did not attenuate ketamine’s psychotomimetic side-effects, nor did it lead to a superior antidepressant response [11]. Similarly, in a recent study by Abdallah et al., pretreatment with lamotrigine did not improve ketamine’s psychomimetic effect, although, it did reduce ketamine-induced global signal regression in the brain [12]. In addition, there have been some concerns of lamotrigine attenuating the anesthetic effects of ketamine and the implications of drug–drug interactions [13]. A recent international expert opinion on the available evidence for ketamine and esketamine in TRD suggested that lamotrigine may theoretically interact with ketamine, but it is uncertain whether ketamine would attenuate the dissociative action of ketamine or ketamine’s efficacy [14]. At the current time, lamotrigine’s impact on ketamine’s efficacy remains uncertain.

In this study, we leveraged the data from our Ketamine Clinic and included consecutive patients with TRD who received serial IV ketamine or IN esketamine treatments with or without lamotrigine therapy to investigate the differences in treatment outcomes.

## 2. Methods

We had two phases in this study.

### 2.1. Historical Cohort Study

In phase 1, we conducted a secondary analysis of our recently published observational study [15] and included consecutive adult patients (≥18 years) with TRD who received up to 6 IV ketamine infusions (0.5 mg/kg based on actual body weight up to 100 kg, over 40–100 min) or up to 8 IN esketamine (56/84 mg) treatments with or without lamotrigine therapy. This study was approved by the Mayo Clinic Institutional Review Board (IRB #20-012789). The included patients received IV ketamine or IN esketamine treatment between 17 August 2017 and 24 June 2021 and provided consent. Details regarding the study methodology were published earlier [15]. The Mayo Clinic Depression Center, Rochester, Minnesota, has a Ketamine Clinic where patients with TRD are eligible to receive IV ketamine or IN esketamine. TRD was defined as a failure to respond to at least two adequate trials of depression treatments (antidepressants, mood stabilizers, or atypical antipsychotic drugs with indication for bipolar depression, electroconvulsive treatment [ECT], or transcranial magnetic stimulation [TMS]) in the current episode of a major depressive disorder (MDD) or bipolar depression episode [16]. We used DSM-5 criteria to make the clinical diagnosis of MDD and bipolar disorder. Patients with active psychosis, active substance use disorder (except nicotine and caffeine), or a recent history of substance use, cognitive disorders, and any other primary (i.e., non-mood disorder) psychiatric disorder were not eligible for ketamine treatment. Data regarding clinical characteristics, medications, and comorbidities were collected. The Clinician-Administered Dissociative States Scale (CADSS) was used to measure the dissociation symptoms [17]. Based on our earlier research work suggesting reduced ketamine response with a concomitant high dose of benzodiazepine [18], we required patients to be on less than 4 mg of lorazepam equivalents prior to initiating ketamine/esketamine treatments, thus, limiting the attenuation of ketamine response due to benzodiazepines.

Patients completed the 16-item Quick Inventory of Depressive Symptomatology Self-Report (QIDS-SR) [19] before and approximately 24 h after ketamine/esketamine treatments. This being a real-world clinical study, most patients continued their medications, including lamotrigine, while concurrently receiving ketamine/esketamine. We did not discontinue or reduce the dose of lamotrigine prior to initiating ketamine/esketamine treatment. Treatment response was defined as ≥50% change (reduction) in the QIDS-SR score and remission as a QIDS-SR score ≤ 5. The percent change in the QIDS-SR scores from the baseline and differences in the CADSS scores between the lamotrigine/non-lamotrigine groups were measured.

### 2.2. Case Series

In phase 2, we reviewed electronic medical records to identify additional patients who received serial ketamine or esketamine treatments and continued lamotrigine therapy, from 25 June 2021 to 31 December 2022, and we reported the combined data. The percent change in QIDS-SR scores from the baseline was measured based on the current lamotrigine dose. We investigated the effect of the lamotrigine dose on response and remission in this sub-cohort.

### 2.3. Statistical Analysis

Continuous variables are reported as means ± SD/median (interquartile range [IQR]) and categorical variables as counts and percentages. Linear Model ANOVA tests were used to test for the differences in the continuous demographic and clinical variables. Pearson’s Chi-squared tests were conducted for differences by categorical demographics, medications, and clinical measures between lamotrigine/non-lamotrigine groups. The repeated measures ANOVA was conducted for differences in changes within infusion on oxygen saturation, blood pressure, heart rate, and in the CADSS score. We assessed for differences between the lamotrigine/non-lamotrigine groups in a percent change in the QIDS-SR scores from the baseline after each treatment, adjusting for the baseline QIDS-SR score, age, sex, BMI, and CADSS score during treatment across the first six treatments. The *p*-values in the results are not adjusted for multiple comparisons.

In a pre-planned analysis, we assessed whether the dosage of lamotrigine contributed to a change in the ketamine’s efficacy among patients who continued lamotrigine. We assessed for effects of low-dose lamotrigine (<100 mg daily dose) versus standard-dose lamotrigine (≥ 100 mg daily dose).

All analyses were performed using R software for statistical computing (version 4.2.0) [20] in RStudio IDE (version 2022.07.2 Build 576) and SAS Studio software (version 3.81, SAS Institute Inc., Cary, NC, USA).

## 3. Results

**Figure 1:**
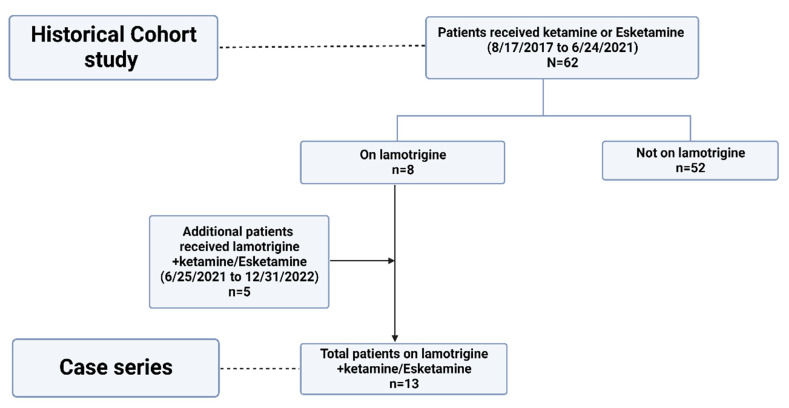
Flow diagram. Created with BioRender.com.

Figure 1 shows the study flow diagram.

### 3.1. Historical Cohort Study

A total of 62 patients received IV ketamine (n = 47) or IN esketamine (n = 15) therapy with a mean age of 47.1 ± 12.1 years and mean BMI of 29.5 ± 6.0, and they were predominantly female (64.5%). Sixty-three percent of patients had comorbid anxiety disorders. Among the 62 patients who had received ketamine or esketamine, eight patients were on continued lamotrigine therapy. Eight patients had received lamotrigine during their current episode of depression but were currently not on lamotrigine (unrelated to ketamine initiation) at the time of ketamine treatments. We did not discontinue lamotrigine for any patient who received ketamine/esketamine. The mean duration of lamotrigine use in patients currently on lamotrigine (at the time of receiving ketamine treatment) versus those who had discontinued lamotrigine (due to any reason) in their current episode of depression was not significantly different (44.5 months vs. 52.2 months, *p* = 0.81). There were no differences in the clinical characteristics, baseline comorbidities, type of antidepressants, stimulants, GABA-positive allosteric modulators, failed neuromodulation treatments (ECT/TMS), response and remission rates, changes within infusion in oxygen saturation, blood pressure, or heart rate as well as in CADSS score among patients treated with or without lamotrigine (Table 1). Compared to patients on lamotrigine, the non-lamotrigine group was on fewer psychotropic medications (4.8 ± 1.0 vs. 3.5 ± 1.4, *p* = 0.02). The response and remission rates were similar to the overall cohort (Table 1).

**Figure 2:**
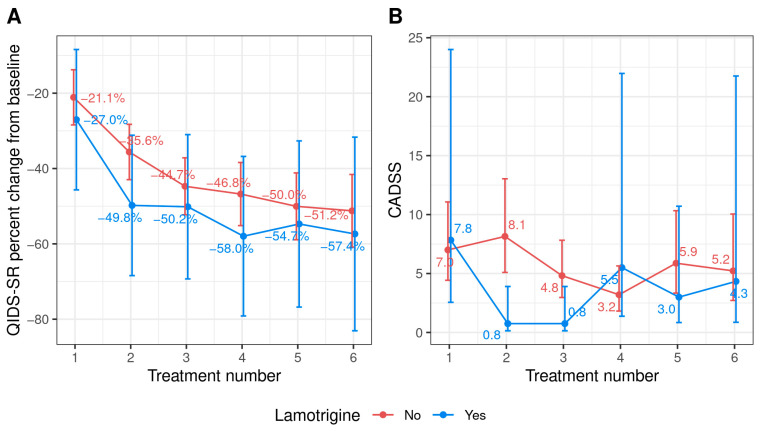
(**A**) Mean percent change in QIDS-SR 16 scores from baseline after each treatment and (**B**) mean CADSS score during treatment based on lamotrigine usage *. Abbreviations—CADSS: Clinician-Administered Dissociative Status Scale; QIDS-SR: 16-item Quick Inventory of Depressive Symptomatology self-report. * n = 48.

For a percent change in QIDS-SR from the baseline, no interaction was found between the lamotrigine groups and treatment number (*p* = 0.70), nor the overall effect of the group (*p* = 0.38) (Figure 2). For the CADSS score, the interaction was not statistically significant (*p* = 0.08), and there was no overall effect on the group (*p* = 0.36). However, when we excluded all the patients receiving esketamine, there was a significant effect of lamotrigine on the CADSS score (*p* = 0.02) but not on the percent change in QIDS-SR from the baseline (*p* = 0.45) among patients receiving IV ketamine. Overall, the CADSS score was lower for those on lamotrigine (across the first five treatments, the mean score was 1.3 vs. 3.8), Supplemental Figure S1A–D.

### 3.2. Case Series

On a further chart review from 25 June 2021 to 31 December 2022, we identified five more patients who received ketamine while continuing lamotrigine therapy. Thus, for phase 2, a total of 13 patients (11 = MDD and 2 = bipolar-II depression) continued lamotrigine therapy and received IV ketamine/IN esketamine treatments during the induction/acute phase. The median age of this sub-cohort (n = 13) was 44 years (IQR, 38–48 years), 85% were females, and the median BMI was 30 kg/m^2^ (IQR, 25–33 kg/m^2^). The detailed clinical characteristics of the patients who continued lamotrigine with ketamine/esketamine are summarized in Table 2. A total of ten patients (77%) received IV ketamine treatments, one patient received IN esketamine, and two patients switched from IV ketamine to IN esketamine after the third IV ketamine infusion, due to financial reasons. Nine patients (69%) responded to the repeated dose regimen, and six (46%) patients met remission criteria at some point during the induction phase. Four patients discontinued treatment due to the lack of an adequate response, and one patient discontinued treatment even after meeting the response criteria as they were dissatisfied with the lack of sustained response. Seven patients continued ketamine/esketamine after achieving a response in the acute phase. Of the six patients who had failed TMS or ECT for depression, 83% (5/6) did not respond to ketamine. Among the non-responders, three patients discontinued after the first three treatment sessions (IV ketamine), and one discontinued after five sessions (IN esketamine). 

**Figure 3:**
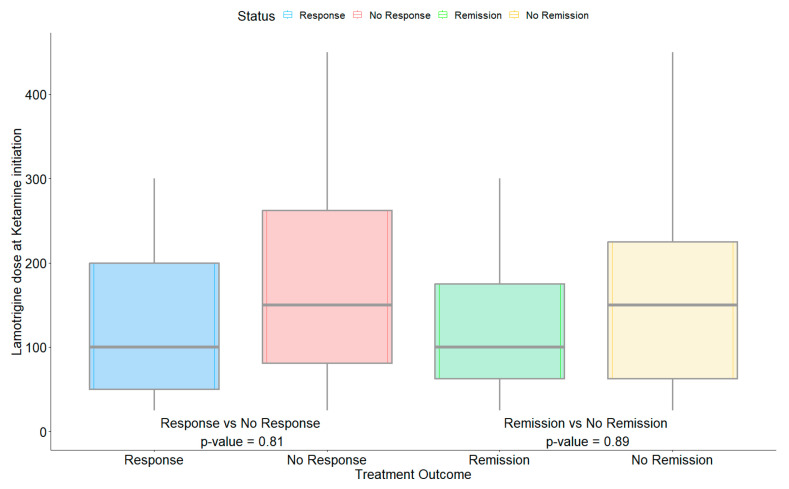
Difference in lamotrigine dose based on the response and remission status at the time of initiating ketamine/esketamine treatments.

Lamotrigine dosing varied from 25 mg to 450 mg, either once daily or twice daily. The median lamotrigine daily dose among the responders and remitters was lower (100 mg daily) than non-responders/non-remitters (150 mg daily), however, the difference was not statistically significant (*p* > 0.81) (Figure 3).

**Figure 4:**
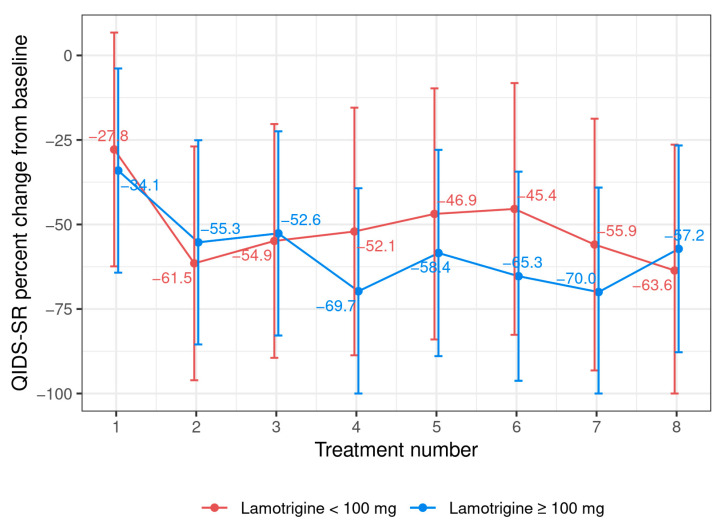
Mean percent change in QIDS-SR 16 scores from baseline after each treatment based on lamotrigine dose.

All the patients were on at least one antidepressant in combination with lamotrigine. Both the patients with bipolar depression who were prescribed lamotrigine responded to ketamine and continued maintenance ketamine treatment after the initial response. We did not notice a significant difference in the percent change in QIDS-SR from the baseline with each ketamine treatment among the patients receiving a low dose of lamotrigine (n = 4) versus the standard dose of lamotrigine (n = 9) (Figure 4).

## 4. Discussion

This preliminary study aimed to investigate and report on the efficacy of serial ketamine/esketamine treatments with or without continued lamotrigine therapy among patients with TRD in a real-world setting. Our study shows insufficient evidence that lamotrigine attenuates the antidepressant response to ketamine treatment. This is an important point considering that it is a common practice for some ketamine clinics to recommend stopping lamotrigine while receiving ketamine treatment with no robust evidence and with the probability of mood destabilization. The response and remission rate with ketamine/esketamine were also similar among patients on lamotrigine or not on lamotrigine [18]. We did not observe a significant difference in the CADSS scores among patients on lamotrigine or not on lamotrigine, although there was a trend towards a lower CADSS score among current lamotrigine users, especially in patients who received IV ketamine. This finding is similar to the study by Anand et al. [9] where lamotrigine administered prior to the ketamine treatment attenuated the psychomimetic effects of ketamine, although the mean lamotrigine dose was much lower in our study. There were significant dose variabilities for lamotrigine among the included cohort. However, there were no significant differences in the lamotrigine dosage among the ketamine responders and non-responders (*p* = 0.8).

Ketamine was overall well tolerated. One patient decided to discontinue IN esketamine due to the lack of effect and emergence of moderate side effects. Off-label IV ketamine is increasingly used by providers for TRD, anxiety, obsessive-compulsive disorder, and post-traumatic stress disorder [21]. Significant and rapid symptom improvements in patients with TRD following a single dose of ketamine treatment are well established, but there are limited data regarding therapeutic interventions that may prolong and/or enhance ketamine’s short-acting effects [5]. Thus, it is important to identify interventions that could enhance or attenuate the ketamine’s response. Current evidence favors repeated ketamine administration for sustained symptom relief [22,23], although there are limited data regarding the durability and efficacy of long-term ketamine use.

Since the discovery of potential antidepressant effects of NMDA receptor antagonists, there has been an increase in evidence favoring the glutamate hypothesis of depression [24]. Imaging studies have reported abnormal glutamate cycling in patients with TRD, distinctively different from non-TRD patients with major depression [25]. Ketamine induces dissociative and psychotomimetic effects in a dose-dependent manner. Dissociative and psychotomimetic effects have also been reported with newer compounds and therapies trialed for potential rapid antidepressant action. However, the dissociative and psychotomimetic effects do not seem to associate with the core antidepressant response to ketamine [26,27]. Mechanistically, the antidepressant effect of ketamine is hypothesized to be due to a preferential inhibition of NMDA receptors expressed on gamma-aminobutyric acid (GABA)/GABAergic interneurons, resulting in reduced inhibitory control over glutamate neurons and subsequent glutamate burst. However, at the same time, some studies have shown that ketamine may attenuate the anterior cingulate cortex GABA deficits among TRD patients who achieved remission status [28,29]. Multiple alternate mechanisms of action have also been proposed, such as enhancing the brain-derived neurotrophic factor release [30], activation of the mammalian target of rapamycin complex-1 [2], and opioid system activation [31]. Moreover, ketamine has also been found to influence diverse astrocyte functions and may also partially exert its antidepressant effect through its effect on astrocytes that modulate neuronal excitability and synaptic transmission [32]. It is considered that the proposed alternate mechanism of actions of ketamine are mutually exclusive, although complement each other in exerting its antidepressant effects [33]. Ketamine’s mechanism of action remains an area of active investigation.

Lamotrigine is an antiepileptic drug, which is also FDA-approved for the maintenance treatment of bipolar disorder [7]. Current guidelines suggest lamotrigine as a well-tolerated second-line augmentation agent for TRD [34]. The effectiveness of lamotrigine augmentation in TRD is attributed to its membrane-stabilizing actions through the inhibition of voltage-sensitive sodium channels and subsequent inhibition of cortical glutamatergic neurotransmission [35]. The effect of lamotrigine augmentation is reported to be more prominent in severely treatment-resistant patients but could also be delayed due to the time needed for dose titration [36,37]. Lamotrigine has a broad clinical efficacy, not all of which is completely explained by its sodium channel-blocking actions [38]. Previous studies have suggested that the reduced glutamate transmission with lamotrigine when compared with the glutamate burst produced with ketamine could constitute a potential antagonistic drug interaction. However, recent studies have reported equivocal data. A pharmacological magnetic resonance imaging study by Doyle et al. reported that lamotrigine administration resulted in attenuation of the BOLD responses to ketamine in the frontal and thalamic regions [39]. Interestingly, Mathew et al. reported that pretreatment with lamotrigine did not enhance the antidepressant effects of a single-dose IV ketamine [11]. Anand et al. reported that in healthy subjects, pretreatment with lamotrigine increased the immediate mood-elevating effects of ketamine while being assessed with Item # 1 of the Young Mania Rating Scale [9]. A few case series and observational studies have reported on concurrent lamotrigine and ketamine use, however, none of those studies systematically investigated the relationship between lamotrigine and ketamine/esketamine [40,41,42]. One study in fact showed an increase in psychomimetic symptomatology (measured using the Brief Psychiatric Rating Scale) with mood stabilizers including lamotrigine (n = 7) after the eighth infusion [42]. These contrasting findings could be due to the small sample size and the use of concurrent medications. Lamotrigine treatment also enhances GABA levels at clinically therapeutic doses [43], thus, potentially reducing glutamate disinhibition in TRD [44]. This complex interaction needs further investigation.

## 5. Limitations

Limitations of this study include, first, the small sample size and patients taking multiple medications. We should also consider the external validity of these results since these data were derived from a specialized mood disorder clinic providing care to significantly TRD patients, which may limit generalizability to non-TRD patients. Second, the population studied was mainly white and adult females, and some patients were on IN esketamine, while others treated were treated with IV ketamine, which may have impacted our findings. However, we had shown a similar response between IV and IN esketamine in this cohort [15]. Third, four patients were on a ≤50 mg dose of lamotrigine, which is much lower than commonly used in clinical practice (100–200 mg). There was a wide range of dosing and no apparent correlation with dose and response. However, the question as to whether higher doses may be more problematic than lower doses need further investigation. Fourth, in our analysis, we did not differentiate between unipolar or bipolar depression, which have different longitudinal courses of illness and clinical responses to ketamine. Three patients discontinued after the first three treatment sessions (IV ketamine), and one discontinued after five sessions (IN esketamine). Our prior data suggest a plateauing effect with IV ketamine treatment at about three infusions [45], thus, we did not see a much more improved response after that [15,45,46]. Thus, it is likely that those patients would have benefited from additional treatments. However, our findings reflect real-world pharmacological interactions in a cohort of TRD patients while receiving ketamine/esketamine treatment.

## 6. Conclusions

In conclusion, this preliminary small sample size study does not suggest that continuation of lamotrigine therapy attenuates the antidepressant effect of repeated ketamine/esketamine treatments. However, there is a trend toward attenuation of dissociation with lamotrigine, especially in patients receiving IV racemic ketamine. These findings need to be investigated in large prospective studies or randomized controlled trials.

## Figures and Tables

**Table 1:** Patient characteristics by ketamine/esketamine with lamotrigine treatment group (no/yes) and overall.

Characteristics	No (n = 54)	Yes (n = 8)	Total (n = 62)	*p* Value
**Age**				0.97 (1)
- Mean (SD)	47.1 (12.3)	47.2 (12.1)	47.1 (12.1)	
- Range	19.9–68.4	29.4–63.1	19.9–68.4	
**Sex (Female)**	34 (63.0%)	6 (75.0%)	40 (64.5%)	0.51 (2)
**BMI**				0.93 (1)
- Mean (SD)	29.5 (6.1)	29.3 (6.1)	29.5 (6.0)	
- Range	20.8–50.9	22.1–39.6	20.8–50.9	
**Employed—Baseline**				0.50 (2)
- Unemployed	2 (3.7%)	1 (12.5%)	3 (4.8%)	
- Employed	30 (55.6%)	3 (37.5%)	33 (53.2%)	
- Disability due to depression	12 (22.2%)	3 (37.5%)	15 (24.2%)	
- Homemaker/retired/student	10 (18.5%)	1 (12.5%)	11 (17.7%)	
**Diagnosis**				0.27 (2)
- MDD	52 (96.3%)	7 (87.5%)	59 (95.2%)	
- Bipolar-II disorder	1 (1.9%)	1 (12.5%)	2 (3.2%)	
- Bipolar-I disorder	1 (1.9%)	0 (0.0%)	1 (1.6%)	
**Duration of depressive episode (years)**				0.47 (1)
- Mean (SD)	5.5 (6.5)	3.9 (3.1)	5.3 (6.1)	
- Range	0.3–37.0	0.8–10.0	0.3–37.0	
**Comorbidities, n (%)**				
PTSD	5 (9.3%)	1 (12.5%)	6 (9.7%)	0.77 (2)
GAD/anxiety disorders	34 (63.0%)	5 (62.5%)	39 (62.9%)	0.98 (2)
Fibromyalgia/chronic pain	6 (11.1%)	1 (12.5%)	7 (11.3%)	0.91 (2)
OCD	2 (3.7%)	0 (0.0%)	2 (3.2%)	0.58 (2)
Eating disorder	3 (5.6%)	0 (0.0%)	3 (4.8%)	0.49 (2)
Borderline personality disorder	5 (9.3%)	0 (0.0%)	5 (8.1%)	0.37 (2)
History of substance use disorder	8 (14.8%)	0 (0.0%)	8 (12.9%)	0.24 (2)
**Intranasal Esketamine**	14 (25.9%)	1 (12.5%)	15 (24.2%)	0.41 (2)
Baseline QIDS-SR				0.47 (1)
- Mean (SD)	17.5 (3.4)	18.5 (3.9)	17.7 (3.5)	
- Range	9.0–24.0	11.0–24.0	9.0–24.0	
**QIDS-SR change from baseline to post-infusion #1**				0.52 (1)
- Mean (SD)	−4.0 (4.6)	−5.1 (4.2)	−4.1 (4.6)	
- Range	−16.0–4.0	−9.0–3.0	−16.0–4.0	
**QIDS-SR percent change from baseline to post-infusion #1**				0.57 (1)
- Mean (SD)	−23.3 (27.2)	−29.1 (26.7)	−24.1 (27.0)	
- Range	−81.2–26.7	−63.6–18.8	−81.2–26.7	
Response	31 (57.4%)	5 (62.5%)	36 (58.1%)	0.79 (2)
Remission	21 (38.9%)	3 (37.5%)	24 (38.7%)	0.94 (2)
CADSS score, mean (SE) ^a^	6.08 (1.06)	3.55 (2.65)	5.73 (0.98)	0.38 (3)
**Change in the vitals**				
Oxygen saturation, mean (SE) ^a^				
Change at 40 min ^b^	0.05 (0.36)	0.61 (0.88)	0.13 (0.33)	0.56 (3)
Change to endpoint ^c^	−0.07 (0.37)	0.63 (0.91)	0.03 (0.34)	0.48 (3)
Systolic BP, mean (SE) ^a^				
Change at 40 min ^b^	5.41 (1.19)	5.52 (2.93)	5.41 (1.09)	0.97 (3)
Change to endpoint ^c^	3.67 (1.22)	5.63 (2.99)	3.94 (1.12)	0.55 (3)
Diastolic BP, mean (SE) ^a^				
Change at 40 min ^b^	2.92 (0.73)	2.19 (1.86)	2.82 (0.67)	0.72 (3)
Change to endpoint ^c^	2.16 (0.76)	0.20 (1.95)	1.90 (0.71)	0.36 (3)
Heart rate, mean (SE) ^a^				
Change at 40 min ^b^	−0.41 (0.82)	0.77 (2.01)	−0.24 (0.76)	0.59 (3)
Change to endpoint ^c^	−1.05 (0.86)	0.46 (2.11)	−0.84 (0.80)	0.51 (3)
**Current # of psychotropics**				0.02 (1)
- Mean (SD)	3.5 (1.4)	4.8 (1.0)	3.7 (1.4)	
- Range	1.0–7.0	3.0–6.0	1.0–7.0	
**Individual psychotropic/class, n (%)**				
SSRI	15 (27.8%)	1 (12.5%)	16 (25.8%)	0.36 (2)
SNRI	18 (33.3%)	3 (37.5%)	21 (33.9%)	0.82 (2)
TCA	7 (13.0%)	1 (12.5%)	8 (12.9%)	0.97 (2)
MAOIs	2 (3.7%)	0 (0.0%)	2 (3.2%)	0.58 (2)
Antipsychotics	15 (27.8%)	5 (62.5%)	20 (32.3%)	0.05 (2)
Mirtazapine	5 (9.3%)	0 (0.0%)	5 (8.1%)	0.37 (2)
Bupropion	14 (25.9%)	2 (25.0%)	16 (25.8%)	0.96 (2)
Stimulant	12 (22.2%)	2 (25.0%)	14 (22.6%)	0.86 (2)
Trazodone	16 (29.6%)	3 (37.5%)	19 (30.6%)	0.65 (2)
Gabapentin	6 (11.1%)	1 (12.5%)	7 (11.3%)	0.91 (2)
GABA-positive allosteric modulators	27 (50.0%)	5 (62.5%)	32 (51.6%)	0.51 (2)
**Neuromodulation (tried in current episode)**				
ECT	16 (29.6%)	3 (37.5%)	19 (30.6%)	0.65 (2)
TMS	8 (14.8%)	2 (25.0%)	10 (16.1%)	0.47 (2)

1. Linear model ANOVA. 2. Pearson’s Chi-squared test. 3. Repeated measures ANOVA. ^a^ n = 40 to 41 for No. of non-Lamotrigine patients because data were missing for 13 to 14 patients. n = 7 for Lamotrigine patients because data were missing for one patient. ^b^ The value closest to 40 min after the start of treatment was used. ^c^ The value at the end of the session within 100 min was used. Abbreviations: BP, blood pressure; CADSS, Clinician-Administered Dissociative States Scale score; ECT, electroconvulsive treatment; GABA-positive allosteric modulators: Gamma-Aminobutyric-Acid-positive allosteric modulators including zaleplon/zolpidem/eszopiclone or low-dose benzodiazepines; MAOIs, monoamine oxidase inhibitors; MDD, major depressive disorder; OCD, obsessive-compulsive disorder; PTSD, post-traumatic stress disorder; SD, standard deviation; SE, standard error; SNRI, serotonin and norepinephrine reuptake inhibitors; SSRI, selective serotonin reuptake inhibitors; TCA, tricyclic antidepressants; TMS, transcranial magnetic stimulation.

**Table 2:** Clinical characteristics and treatment outcomes of patients on lamotrigine.

Patient	Age	Sex	BMI	Current Psychiatric Diagnosis	Concurrent Psychiatric Medications	Duration of Current Depressive Episode	Failed Interventions in Current Episode	IV Ketamine/ In Esketamine	Baseline QIDS-SR 16 Score	Response (Yes/No)	Remission (Yes/No)	Continued Ketamine/Esketamine after the Acute Phase
1.	44	F	21.32	MDD, GAD	Aripiprazole (2 mg/day), Venlafaxine-XR (375 mg/day), Lamotrigine (250 mg/day), Clonazepam (0.5 mg TID)	5 years	Citalopram	IV ketamine	19	Yes	Yes	Yes
2.	61	F	33.27	MDD, PTSD	Lurasidone (20 mg/day), Trazodone (75 mg/day), Lamotrigine (25 mg/day), Vilazodone (35 mg/day)	10 years	Venlafaxine XR, Bupropion XL, Modafinil, Aripiprazole	IV, switched to IN esketamine after 3 treatments	20	Yes	No	Yes—Esketamine
3.	63	M	39.93	BD-II, GAD	Bupropion (150 mg/day), Vortioxetine (10 mg/day), Lamotrigine (100 mg/day), Dextroamphetamine–Amphetamine (20 mg/day), Lorazepam (2 mg/day)	5 years	Lithium, Venlafaxine, Quetiapine, Lurasidone, Brexpiprazole, Valproate, Carbamazepine, Olanzapine, Aripiprazole, Fluoxetine, Citalopram, Escitalopram, Sertraline, Nefazodone, Duloxetine, Vilazodone, Tranylcypromine, Phenelzine, Imipramine, ECT, TMS	IV ketamine	15	Yes	Yes	Yes
4.	54	F	27.98	MDD, PTSD	Aripiprazole (5 mg/day), Duloxetine (120 mg/day), Lamotrigine (100 mg/day), Alprazolam (1.5 mg/day)	5 years	Fluoxetine, Trazodone, Modafinil, Bupropion	IV ketamine	18	Yes	Yes	Yes
5.	36	F	27.05	MDD, GAD	Nortriptyline (50 mg/day), Trazodone (50 mg/day), Lamotrigine (150 mg/day), Clonazepam (0.5 mg BID), Gabapentin (300 mg/day), Melatonin (10 mg/day)	1 year	Bupropion, Duloxetine, ECT, Quetiapine	IV ketamine	22	Yes	No	No. Lost effectiveness after discontinuing nortriptyline (due to mild weight gain)
6.	38	M	33.3	MDD, OCD, GAD, Personality disorder	Fluvoxamine (300 mg/day), Brexpiprazole (3 mg/day), Lamotrigine (25 mg/day)	2 years	ECT, Dextroamphetamine–Amphetamine, Bupropion, Clomipramine, Lorazepam	IV ketamine	16	No	No	No. Lack of response
7.	29	F	29.57	MDD, GAD, ADHD	Bupropion-XL (300 mg/day), Lamotrigine (125 mg BID), Quetiapine (25 mg/day), Dextroamphetamine-Amphetamine (25 mg/day)	2 years	Sertraline, Melatonin	IV ketamine	24	Yes	No	Yes. Moved out of the state.
8.	48	F	22.11	MDD, GAD	Iloperidone (3 mg/day), Vilazodone (10 mg/day), Lamotrigine (225 mg BID), Clonazepam (1 mg/day), Trazodone (100 mg/day), Buspirone (30 mg/day), clonidine 0.2 mg QHS	0.8 years	TMS, IM ketamine	IV ketamine	18	No	No	No—Lack of response
9.	45	F	18.36	MDD	Venlafaxine (31.25 mg/day), Sertraline (150 mg/day), Lamotrigine (25 mg/day)	3 years	Aripiprazole, Quetiapine, Cariprazine, Lithium	IV ketamine	15	Yes	Yes	Yes
10.	23	F	33.4	MDD with Anxious Distress	Vortioxetine (10 mg/day), Bupropion (150 mg/day), Lamotrigine (200 mg/day)	1.25 years	Venlafaxine, Duloxetine, TMS, ECT, Quetiapine, Fluvoxamine	IN esketamine	21	No	No	No. Lack of response. Had side effects—nausea, vomiting, dissociations. Discontinued after 5 treatments
11.	43	F	30	BD-II, GAD, Alcohol use disorder, in sustained remission	Cariprazine (3 mg/day), Duloxetine (80 mg/day), Lamotrigine (100 mg QAM, 200 mg QHS), Gabapentin (1200 mg TID)	4.5 years	Lithium, Quetiapine, Lurasidone, Buspirone, Olanzapine, Dextroamphetamine-Amphetamine	IV ketamine	23	Yes	Yes	Yes
12.	43	F	25.23	MDD, GAD, Borderline Personality Disorder	Fluoxetine (20 mg/day), Bupropion-XL (450 mg/day), Buspirone (60 mg/day), Lamotrigine (100 mg/day), Trazodone (100 mg/day), Hydroxyzine (25–50 mg TID)	17 years	Venlafaxine, Duloxetine, Lisdexamfetamine, Risperidone	IV ketamine	15	No	No	No. Lack of response. Discontinued all the psychotropics after 2 treatments.
13.	46	F	33	MDD, GAD	Clonazepam (2 mg/day), Pramipexole (0.5 mg/day), Lamotrigine (50 mg/day), Protriptyline (45 mg/day)	10 years	Aripiprazole, Duloxetine, Venlafaxine, Bupropion, Buspirone, Trazodone, Vortioxetine, Oxcarbazepine, Selegiline, Lithium, ECT	IV, switched to IN esketamine after 3 treatments	22	Yes	Yes	No. Initial response with IV ketamine. Switched to esketamine but did not find prolonged benefit.

Abbreviations: ADHD: attention deficit hyperactive disorder; BD-II: bipolar disorder type II; BID: twice a day; ECT: electroconvulsive therapy; GAD: Generalized Anxiety Disorder; MDD: major depressive disorder; OCD: obsessive-compulsive disorder; PTSD: post-traumatic stress disorder; TID: three times a day; TMS: transcranial magnetic stimulation. Response was defined as ≥50% reduction in the QIDS score from baseline; remission was defined as QIDS-SR ≤ 5.

## Data Availability

The data presented in this study are available on request from the corresponding author. Additional data are not publicly available due to privacy and ethical restrictions.

## Supplementary Materials

The following supporting information can be downloaded at https://www.mdpi.com/article/10.3390/ph16081164/s1, Figure S1: Among patients receiving IV ketamine based on lamotrigine usage: Mean percent change in QIDS-SR 16 scores from baseline (A) after each treatment and (B) overall (n = 47). Mean CADSS score (C) during treatment and (D) overall (n = 36).
