# Correction: Joseph et al. Efficacy of Ketamine with and without Lamotrigine in Treatment-Resistant Depression: A Preliminary Report. *Pharmaceuticals* 2023, *16*, 1164

**DOI:** 10.3390/ph16111571

**Published:** 2023-11-07

**Authors:** Boney Joseph, Nicolas A. Nunez, Simon Kung, Jennifer L. Vande Voort, Vanessa K. Pazdernik, Kathryn M. Schak, Stacey M. Boehm, Brooke Carpenter, Emily K. Johnson, Grigoriy Malyshev, Nathan Smits, Daniel O. Adewunmi, Sarah K. Brown, Balwinder Singh

**Affiliations:** 1 Department of Psychiatry & Psychology, Mayo Clinic, Rochester, MN 55905, USA; 2 Department of Neurology, Mayo Clinic, Rochester, MN 55905, USA; 3 Department of Quantitative Health Sciences, Mayo Clinic, Rochester, MN 55905, USA

## Error in Figure

In the original publication [1], there were mistakes in the Figure 2B. The correct Figure 2B appear below. The authors state that the scientific conclusions are unaffected. This correction was approved by the Academic Editor. The original publication has also been updated.

## Figures and Tables

**Figure 2:**
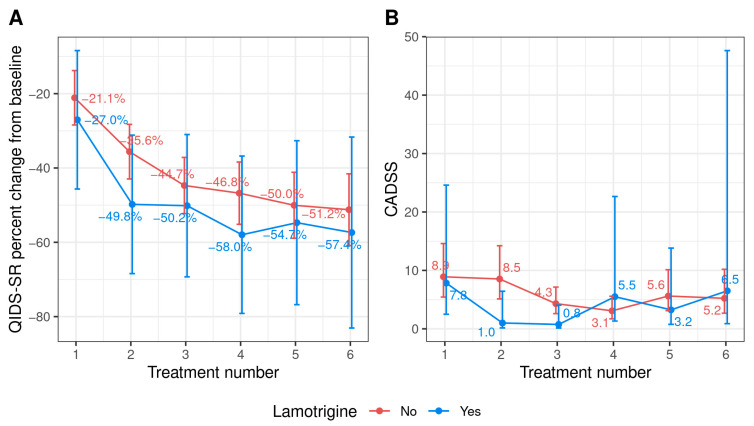
(**A**) Mean percent change in QIDS-SR 16 scores from baseline after each treatment and (**B**) mean CADSS score during treatment based on lamotrigine usage (N = 48). Abbreviations—CADSS: Clinician-Administered Dissociative Status Scale; QIDS-SR: 16-item Quick Inventory of Depressive Symptomatology self-report.
