# Supplementary material for: Efficacy of Ketamine with and without Lamotrigine in Treatment-Resistant Depression: A Preliminary Report
Source: Pharmaceuticals (Basel). 2023 Aug 16;16(8):1164. doi: 10.3390/ph16081164 (PMC10459873; doi:10.3390/ph16081164)
Supplement: Supplementary file 1 [file pharmaceuticals-16-01164-s001.zip › pharmaceuticals-2442363-supplementary.pdf]

Supplement Figure 1. Among patients receiving IV ketamine based on lamotrigine usage: Mean percent change in QIDS-SR 16 scores from baseline (A) after each treatment and (B) overall (N=47). Mean CADSS score (C) during treatment and (D) overall (N = 36).

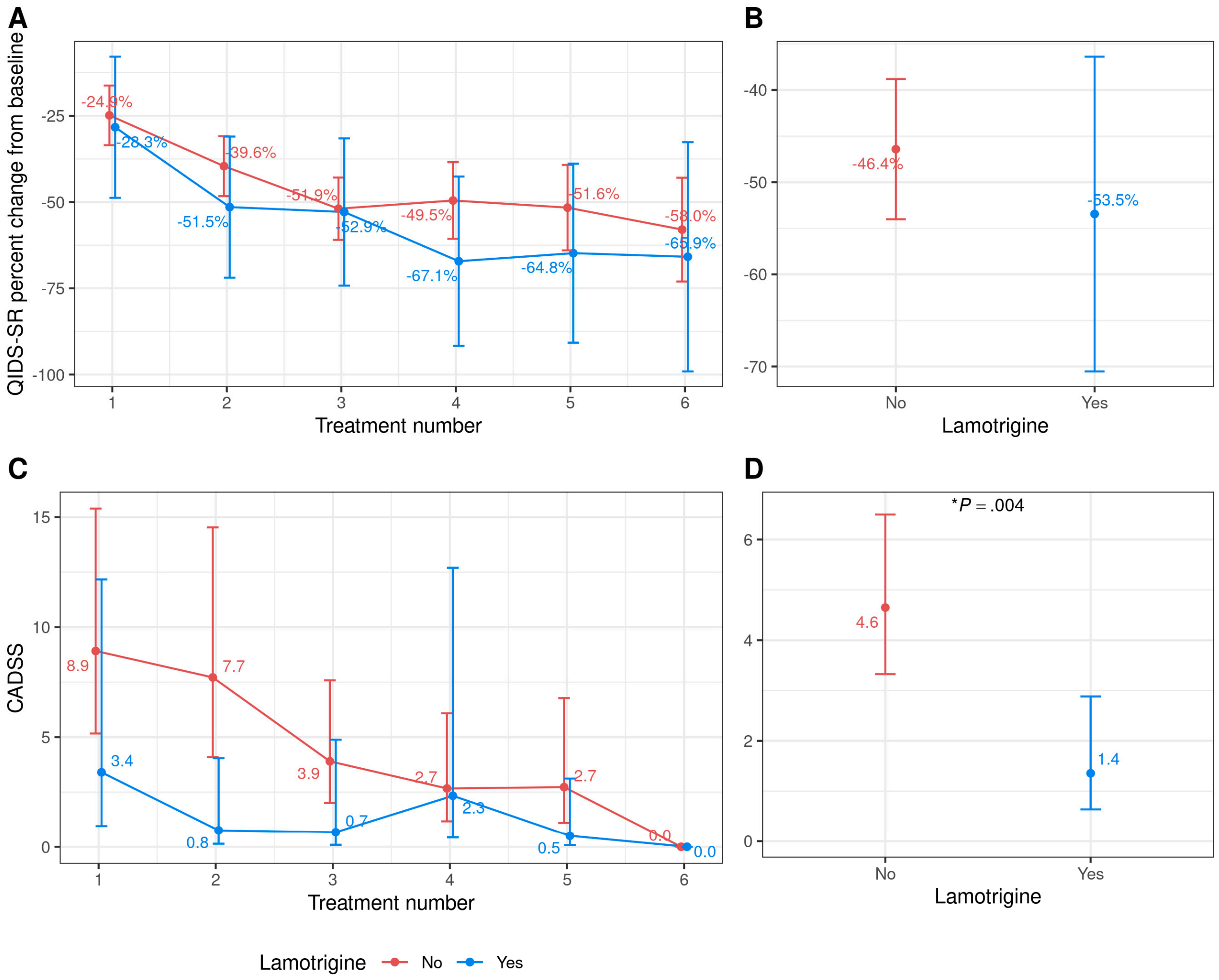

Supplement Figure 1C: No confidence intervals reported for CADSS during treatment number 6 because no variability within either Lamotrigine group.
